# Supplementary material for: TastepepAI: An artificial intelligence platform for taste peptide de novo design
Source: PLoS Comput Biol. 2025 Oct 16;21(10):e1013602. doi: 10.1371/journal.pcbi.1013602 (PMC12543283; doi:10.1371/journal.pcbi.1013602)
Supplement: S1 File — (ZIP) [file pcbi.1013602.s006.zip › Supplementary Data 2/TaPep69 HPLC.doc]

**HPLC REPORT**

| Sample: | NJP93376 TaPep69 AE-5 | | | Analyzed date: | 2024-9-25 |
| --- | --- | --- | --- | --- | --- |
| Analyst: | LY Reconstitution: 1mg/0.5ml H_2_O | | | | |
| Lot. No.: | P240920-MX1172305 | | | | |
| Column: | 250*4.6mm,ChromCore 120 C18 5um | | | | |
| Solvent A | A: 0.1% Trifluoroacetic Acid in 100% Acetonitrile | | | | |
| Solvent B | B: 0.1% Trifluoroacetic Acid in 100% Water | | | | |
| Gradient: |  | A | B |  | |
|  | 0.0min | 8% | 92% |  | |
|  | 25.0min | 33% | 67% |  | |
|  | 25.1min | 100% | 0% |  | |
|  | 30.0min | Stop | |  | |
| Volume: | 10μl | | |  | |
| Wavelength: | 220nm | | |  | |
| Flow rate: | 1.0ml/min | | |  | |
| ───────────────────────────────────────────────────  Peak No Ret Time Height Area Conc.  ───────────────────────────────────────────────────  1 5.818 1189.459 13623.000 0.2329  2 6.665 5897.486 67014.695 1.1457  3 7.107 6946.074 30570.682 0.5227  4 7.295 463324.281 5656034.000 96.7011  5 7.862 4295.498 72078.844 1.2323  6 8.663 1225.285 8823.600 0.1509  7 12.468 96.787 840.250 0.0144  ───────────────────────────────────────────────────  Total 100.00 | | | | | |
